# Association of Polypharmacy and Bone Mineral Density: A Cross-Sectional Analysis of Geriatric Inpatients in Germany

**DOI:** 10.3390/jcm15031197

**Published:** 2026-02-03

**Authors:** Stylianos Kopanos, Sandra Nicole Scheel, Bettina Eggert, Ulrich Thiem, Joachim Feldkamp

**Affiliations:** 1 Academic Department of Endocrinology, Diabetes and Infectiology, Klinikum Bielefeld, Medical School, University Medical Centre East Westphalia-Lippe Bielefeld University, 33617 Bielefeld, Germany; sandra.scheel@klinikumbielefeld.de (S.N.S.); bettina.eggert@klinikumbielefeld.de (B.E.); joachim.feldkamp@klinikumbielefeld.de (J.F.); 2 Department of Geriatrics, Medical School Ostwestfalen-Lippe (OWL), University of Bielefeld, 33647 Bielefeld, Germany; ulrich.thiem@klinikumbielefeld.de

**Keywords:** osteoporosis, bone mineral density, polypharmacy, frailty, multimorbidity

## Abstract

**Background:** Osteoporosis is a prevalent metabolic bone disorder characterized by reduced bone mineral density (BMD) and increased fracture risk, particularly among older adults. While individual medications have been implicated in bone loss, the cumulative impact of polypharmacy on skeletal health remains underexplored. **Methods:** This cross-sectional study included 1155 geriatric inpatients undergoing routine bone mineral density assessment. Medication use, demographic characteristics, and clinical variables were extracted from electronic medical records. BMD at the lumbar spine (L1–L4) and total hip was measured using dual-energy X-ray absorptiometry (DXA). Unadjusted analyses and multivariable linear regression models were used to examine associations between medication use, polypharmacy (defined as the use of ≥5 medications), and BMD, adjusting for age, sex, body mass index, and relevant clinical covariates. **Results:** The mean age of the study population was 85.0 ± 7.1 years, and 80.1% were female. Polypharmacy was present in 64.5% of patients. In medication-specific analyses, thyroid hormone use was associated with lower lumbar spine BMD (*p* = 0.032), and concomitant use of diuretics and proton pump inhibitors was associated with lower hip BMD (*p* = 0.049). Steroid use showed a marginally non-significant correlation with reduced BMD (*p* = 0.057). Polypharmacy was associated with lower lumbar spine BMD (*p* = 0.022), whereas no significant association was observed with hip BMD. Increasing age was consistently associated with lower BMD across skeletal sites (*p* < 0.001). **Conclusions:** In this geriatric inpatient cohort, polypharmacy and selected medication classes were associated with lower bone mineral density, particularly at the lumbar spine. Given the cross-sectional design, these findings reflect associations rather than causal relationships and may partly capture underlying multimorbidity and clinical complexity. Consideration of medication burden may be relevant when evaluating bone health in older adults. Polypharmacy is increasingly common in older adults and may contribute to bone fragility. In this cohort of 1155 geriatric inpatients, multiple medications and certain drug classes were associated with lower bone mineral density, particularly in the spine. These findings suggest that medication burden should be considered when evaluating osteoporosis risk in aging populations.

## 1. Introduction

Osteoporosis is a prevalent metabolic bone disorder characterized by reduced bone mineral density (BMD) and an increased susceptibility to fractures, posing a significant public health burden, particularly among older adults. The condition results from an imbalance between bone resorption and formation, influenced by a combination of genetic, hormonal, lifestyle, and pharmacological factors [1,2]. Among these, medication use has emerged as a crucial yet often overlooked contributor to bone loss, with certain drug classes being associated with increased bone turnover and heightened fracture risk [3].

Several widely prescribed medications, including L-thyroxine, diuretics, proton pump inhibitors (PPIs), and corticosteroids, have been associated with BMD reduction and increased osteoporosis risk [4]. Corticosteroids, for instance, are well-documented potent inducers of bone resorption, leading to secondary osteoporosis, while long-term PPI use has been linked to impaired calcium absorption, contributing to compromised skeletal integrity. The role of thyroid hormone replacement therapy (L-thyroxine) in osteoporosis remains a subject of debate, with some evidence suggesting that excessive doses may contribute to increased bone turnover [5]. Additionally, the impact of antidepressants and anti-inflammatory agents on bone metabolism remains controversial, with inconsistent findings regarding their role in osteoporosis development [6].

Despite the growing recognition of medication-induced osteoporosis, existing research primarily focuses on the effects of individual drugs rather than the cumulative impact of polypharmacy on bone health [7,8]. Given the increasing prevalence of polypharmacy in aging populations, it is crucial to assess how the combined use of multiple medications may contribute to osteoporosis risk and fracture susceptibility [9,10].

In geriatric populations, polypharmacy is closely intertwined with frailty and functional decline, both of which are independently associated with low bone mineral density and fracture risk. Understanding polypharmacy-related bone outcomes therefore requires consideration of frailty as a key component of clinical complexity in older adults. This study aims to provide a comprehensive evaluation of the association between multiple medication use and osteoporosis risk, with a specific focus on the association between polypharmacy and bone mineral density. By investigating how various drug classes influence skeletal health, this research seeks to enhance clinical awareness and inform evidence-based strategies for osteoporosis prevention and management, ultimately contributing to improved patient outcomes in individuals exposed to long-term pharmacological therapies.

## 2. Materials and Methods

### 2.1. Study Design and Population

This was a single-centre, cross-sectional observational study conducted in a diverse geriatric inpatient population. The study was designed to examine cross-sectional associations between polypharmacy and bone mineral density. Data were sourced from individuals who underwent routine BMD assessments at our specialized endocrinology and osteoporosis referral centre, ensuring a well-characterized cohort with comprehensive medical documentation.

### 2.2. Data Collection and Patient Selection

Patients were recruited from a geriatric inpatient unit of a tertiary care hospital in Germany. Bone mineral density measurements were performed as part of routine inpatient diagnostic evaluation at the affiliated endocrinology and osteoporosis service during hospital admission. Electronic medical records were systematically reviewed to extract relevant clinical, demographic, and pharmacological data. The study population included patients with documented BMD measurements, ensuring the availability of objective skeletal health assessments. Only individuals with detailed medication histories were considered, allowing for an accurate evaluation of the potential effects of single-drug use and polypharmacy on bone health.

Key demographic variables, such as age, sex, body mass index (BMI), metabolic profile variables and comorbidities were collected to account for potential confounders. Clinical history was thoroughly reviewed, capturing osteoporosis risk factors, prior fractures, history of falls, and chronic disease profiles, ensuring a comprehensive assessment of factors influencing BMD.

Medication data were extracted from electronic medical records at the time of hospital admission and reflected regularly prescribed medications documented during inpatient medication reconciliation. For most medication classes evaluated in this study (e.g., proton pump inhibitors, diuretics, thyroid hormone replacement, glucocorticoids), current use at admission is routinely and reliably recorded, as these drugs are prescribed continuously and require active inpatient continuation or withholding.

In contrast, osteoporosis-specific pharmacological treatments (e.g., bisphosphonates, denosumab, teriparatide) are typically administered intermittently, often initiated in outpatient settings, and may not be actively prescribed or continued during acute inpatient stays. As a result, documentation in inpatient electronic medical records frequently reflects historical or incomplete treatment information without reliable data on timing, duration, or recency of exposure. Consequently, osteoporosis-specific pharmacotherapy could not be consistently or temporally classified and was therefore not included in descriptive tables or analyses.

As a result, medication exposure was treated as a binary variable (current use vs. non-use) at the time of BMD assessment. Although functional status and comorbidity data were routinely documented, a standardized frailty score was not systematically recorded and could not reliably be reconstructed retrospectively for the entire cohort.

The study was conducted in accordance with the Declaration of Helsinki and approved by the local ethics committee (approval number: approval number 2025-443-f-S). Due to the retrospective nature of the study and use of anonymized routine clinical data, the requirement for informed consent was waived.

### 2.3. Inclusion and Exclusion Criteria

All eligible patients admitted between January 2018 and December 2023 were screened for inclusion. To enhance the study’s validity and generalizability, specific inclusion and exclusion criteria were applied. Patients were eligible if they had complete baseline BMD assessments and documented laboratory and medication data. The analysis included individuals prescribed medications commonly implicated in bone metabolism alterations, such as thyroid hormones, proton pump inhibitors (PPIs), glucocorticoids, diuretics, NSAIDs, and antidepressants.

Polypharmacy was defined as the concurrent use of five or more prescribed medications, in accordance with definitions commonly used in geriatric populations. Although the most widely cited definition of polypharmacy is ≥5 medications, recent systematic reviews highlight substantial variability in the literature, with thresholds ranging from ≥2 to ≥11 drugs [3]. Our threshold (≥5) reflects the advanced age and high multimorbidity burden of our cohort. This threshold was chosen in accordance with commonly used geriatric definitions of polypharmacy and is widely applied in studies involving older and multimorbid populations. A cut-off of ≥5 medications remains the most frequently used and clinically relevant threshold in geriatric research, particularly in very old inpatient cohort [11,12].

Patients were excluded if they had incomplete medical records or severe medical conditions that could independently impact BMD, such as end-stage renal disease (eGFR <30 mL/min/1.73 m^2^), active malignancies with bone metastases, or chronic inflammatory diseases affecting skeletal integrity (e.g., untreated rheumatoid arthritis or systemic lupus erythematosus). Additionally, individuals receiving long-term high-dose corticosteroid therapy (>5 mg prednisolone equivalent for more than six months) or those with uncontrolled endocrine disorders (e.g., untreated hyperthyroidism or hyperparathyroidism) were excluded to minimize confounding effects on bone metabolism. A detailed flow diagram illustrating patient identification, exclusions, and the final analytical sample is provided in Figure S1, in accordance with STROBE recommendations.

By applying a rigorous selection process, this study aimed to provide a detailed and clinically relevant evaluation of how polypharmacy and specific medication classes influence osteoporosis risk and bone health outcomes in a real-world clinical setting.

Patients were not excluded on the basis of prior or ongoing osteoporosis-specific pharmacological treatment. Given the advanced age, high multimorbidity burden, and long treatment histories of this geriatric inpatient cohort, reliable identification and temporal classification of prior osteoporosis therapy was not feasible using routinely collected electronic medical records. Excluding all patients with any history of osteoporosis treatment would have resulted in substantial loss of sample size and reduced representativeness of real-world geriatric care. Calcium and vitamin D supplementation were not considered osteoporosis-specific treatments and were therefore not exclusion criteria. These supplements were common in the cohort and reflect routine clinical practice in geriatric inpatient populations.

The study is reported in accordance with the Strengthening the Reporting of Observational Studies in Epidemiology (STROBE) guidelines (Supplementary Table S1).

### 2.4. Bone Mineral Density Assessment

BMD was evaluated at two key anatomical sites, the hip and lumbar spine, using dual-energy X-ray absorptiometry (DXA) with a Lunar iDXA system (GE Healthcare, Madison, WI, USA), a gold-standard technique for osteoporosis diagnosis. Osteoporosis was classified according to the World Health Organization (WHO) criteria, with a T-score of ≤−2.5 standard deviations (SDs) below the young adult reference range indicating osteoporosis. All measurements were performed at a single tertiary referral center using a standardized clinical protocol. DXA examinations were conducted by trained and certified technicians according to manufacturer recommendations.

DXA scans were reviewed as part of routine clinical reporting; however, cohort-specific precision error metrics (coefficient of variation, CV%) and least significant change (LSC) values could not be calculated retrospectively, and systematic exclusion of degenerative changes, vascular calcifications, or vertebral deformities was not feasible. Consequently, the magnitude of measurement variability cannot be fully quantified, particularly at the lumbar spine, where precision errors are typically higher, and this should be considered when interpreting site-specific BMD associations.

### 2.5. Statistical Analysis

Statistical analysis was performed to examine cross-sectional associations between polypharmacy, medication use, and bone mineral density (BMD). Continuous variables are presented as means with standard deviations or medians with interquartile ranges, as appropriate, while categorical variables are reported as frequencies and percentages.

Medication-use patterns were described to characterize the prevalence of individual drug classes and the overall burden of polypharmacy within the study population. Polypharmacy was defined a priori as the concurrent use of five or more regularly prescribed medications at the time of hospital admission. The effects of specific drug categories on BMD were assessed using independent *t*-tests, comparing mean BMD values at the hip and lumbar spine in users versus non-users of the following medications: Thyroid hormones (e.g., L-Thyroxin), Proton pump inhibitors (PPIs), Glucocorticoids (systemic and inhaled steroids), Diuretics, analysed both as a broad category and further stratified into thiazide diuretics, loop diuretics, and potassium-sparing diuretics, Nonsteroidal anti-inflammatory drugs (NSAIDs) and Antidepressants (both Selective Serotonin Reuptake Inhibitors (SSRIs) and Serotonin–Norepinephrine Reuptake Inhibitors (SNRIs).

Unadjusted comparisons of BMD values at the lumbar spine (L1–L4) and total hip were conducted using independent *t*-tests or analysis of variance (ANOVA), as appropriate, to explore differences between medication users and non-users and across categories of medication burden. Serum 25-hydroxyvitamin D levels were available in a subset of patients and were included as a covariate where measured. Analyses were performed using a complete-case approach, and no imputation of missing laboratory values was undertaken.

To account for potential confounding, multivariable linear regression models were constructed with BMD at the lumbar spine and total hip as continuous dependent variables. Polypharmacy status (≥5 medications) was included as the primary independent variable. Covariates were selected a priori based on clinical relevance and existing literature and included age, sex, body mass index (BMI), glucocorticoid use, chronic kidney disease, diabetes mellitus, smoking status, and vitamin D status, where available.

Model assumptions were assessed using standard diagnostic procedures. Results are presented as regression coefficients with 95% confidence intervals. In sensitivity analyses, polypharmacy was additionally modelled as a continuous variable (number of medications) to evaluate the robustness of the observed associations.

Comorbidities were included as individual covariates in multivariable models to account for major disease-related confounding. However, formal multimorbidity clustering or pattern analyses were not performed due to the retrospective design and heterogeneity of diagnoses in this very old inpatient cohort.

Given the cross-sectional nature of the study and the limitations of routinely collected electronic medical records, analyses focused on current medication exposure rather than cumulative duration. Sensitivity analyses stratified by exposure duration were not feasible due to incomplete and heterogeneous documentation of treatment history. No stratification or adjustment based on prior osteoporosis-specific pharmacological treatment was performed, as detailed treatment histories were not consistently available across the cohort.

All statistical analyses were performed using RStudio (version 2024.12.0+467, Posit Software, Boston, MA, USA). A two-sided *p*-value < 0.05 was considered statistically significant.

## 3. Results

The study cohort consisted of 1155 patients, with a mean age of 85.0 ± 7.1 years. The majority of participants were female (80.1%). Mean age was 85.4 ± 6.4 years in females and 83.9 ± 7.3 years in males. Baseline characteristics stratified by polypharmacy status are presented in Table 1.

**Figure 1:**
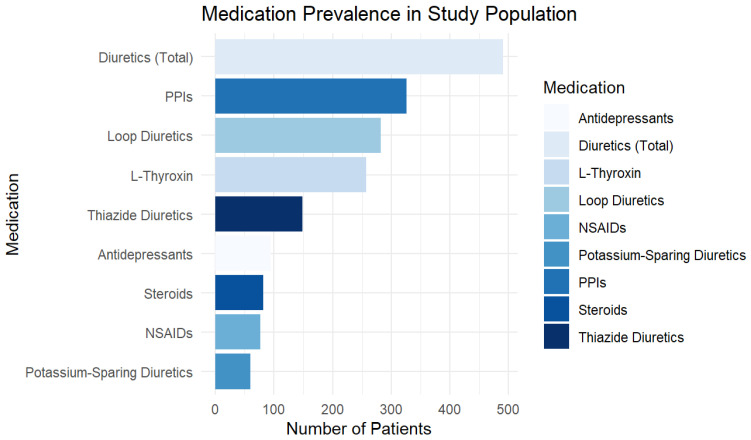
Medication Prevalence in the Study Population (Bar Chart). This bar chart represents the distribution of medications used among study participants. Diuretics are the most commonly prescribed drugs, followed by PPIs and thyroid medications.

Medication use was highly prevalent in the study population. L-Thyroxin was prescribed to 22.3% of patients (n = 257), PPIs to 28.2% (n = 326), and steroids to 7.1% (n = 82). Diuretics were the most frequently used medication category, with 42.5% (n = 491) of patients taking at least one type. Among these, 24.4% (n = 282) were on loop diuretics, 12.9% (n = 149) on thiazide diuretics, and 5.2% (n = 60) on potassium-sparing diuretics. The use of NSAIDs was recorded in 6.7% (n = 77) of patients, while 8.2% (n = 95) were taking antidepressants. Figure 1 provides a graphical representation of medication prevalence, highlighting diuretics, PPIs, and thyroid medications as the most commonly used drug classes.

**Figure 2:**
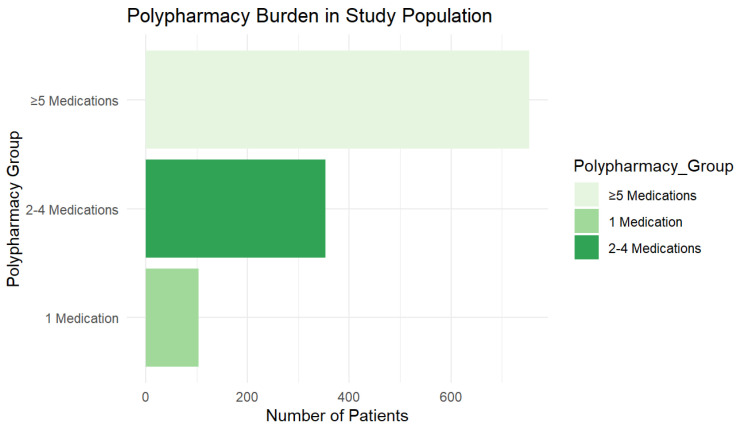
Polypharmacy Burden in the Study Population (Bar Chart). This chart illustrates the extent of polypharmacy within the study population. The majority of patients (64.5%) are taking five or more medications, 30.6% are on 2–4 medications, while only a small proportion (9.0%) are on a single drug.

**Figure 3:**
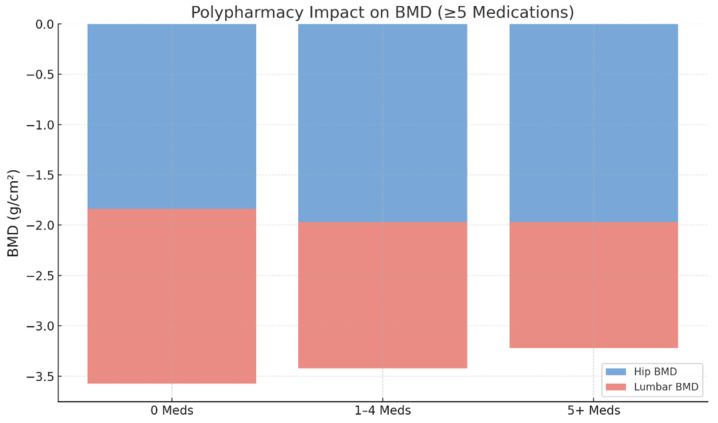
Polypharmacy Impact on BMD. This stacked bar chart illustrates the impact of polypharmacy on bone mineral density (BMD) at the hip and lumbar spine. As the number of medications increases from 0 to 5 or more, there is a noticeable decline in both hip BMD (blue) and lumbar BMD (red). The reduction is more pronounced in the lumbar spine, suggesting that polypharmacy disproportionately affects trabecular bone, which is more metabolically active and susceptible to medication-induced bone loss.

Polypharmacy, defined as the concurrent use of five or more medications, was present in 64.5% (n = 745) of patients, while 30.6% (n = 353) were using two to four medications and 9.0% (n = 104) were using one medication. The distribution of medication burden across the study population is illustrated in Figure 2 and Figure 3, emphasizing the high prevalence of multiple drug use among older adults undergoing osteoporosis assessments.

### 3.1. Associations Between Medication Use and Bone Mineral Density (BMD)

**Figure 4:**
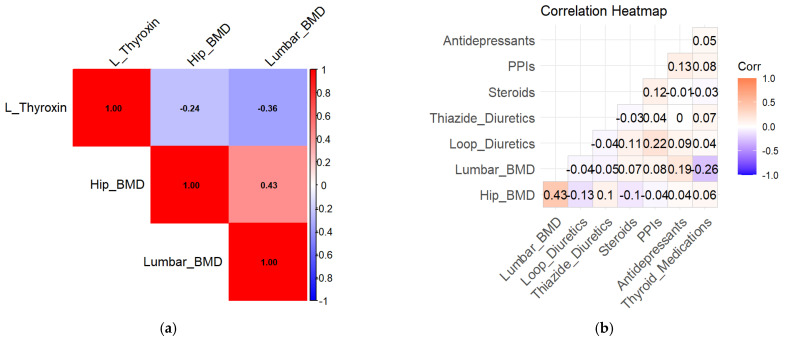
(**a**). Correlation Heatmap for L-Thyroxin and Bone Mineral Density (BMD). Exploratory correlation matrix depicting pairwise correlations between medication use and bone mineral density measurements. (**b**). Comprehensive Correlation Heatmap for Medications and BMD. This heatmap visualizes the relationships between various medications and BMD. The strongest correlation is observed between hip and lumbar BMD (0.43). Negative correlations, though weak, are seen between PPIs, steroids, loop diuretics, and BMD, highlighting the potential adverse effects of these medications on bone health.

In unadjusted analyses, L-thyroxin use was associated with lower lumbar spine BMD (β = −1.45, 95% CI −2.41 to −0.49, *p* = 0.032), indicating a potential adverse effect of thyroid hormone therapy on bone health. Exploratory correlation analyses are shown in Figure 4a.

Concomitant use of diuretics and proton pump inhibitors was associated with lower hip BMD (β = −1.82, 95% CI −3.10 to −0.54, *p* = 0.049). Steroid use was associated with lower BMD values, although this association did not reach statistical significance (β = −1.35, 95% CI −2.45 to −0.25, *p* = 0.057).

**Figure 5:**
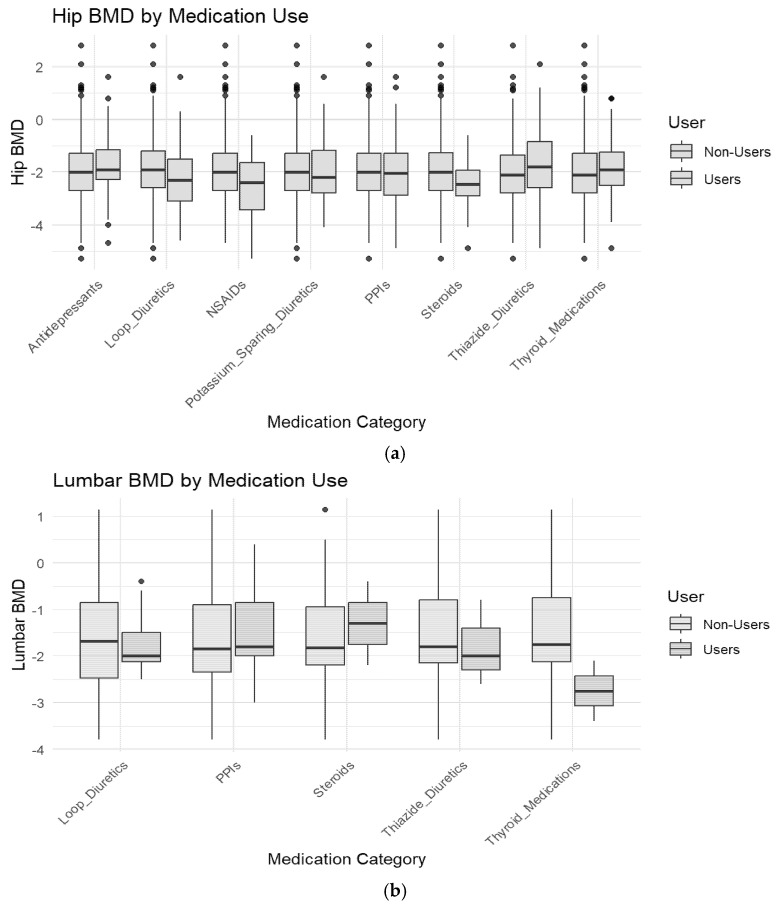
(**a**). Hip BMD by Medication Use (Boxplot). This boxplot compares hip BMD values between medication users and non-users across different drug categories. Users of steroids, loop diuretics and PPIs tend to have lower hip BMD than non-users, suggesting a potential association between these medications and bone loss. (**b**). Lumbar BMD by Medication Use (Boxplot). Similarly to the previous plot, this boxplot displays lumbar BMD values by medication category. A noticeable trend of lower lumbar BMD is seen among users of loop diuretics, steroids, and thyroid medications, reinforcing their suspected role in increasing osteoporosis risk.

Medication-specific BMD distributions are illustrated in Figure 4a,b. Lower BMD values were observed among medication users compared with non-users in unadjusted analyses (Figure 5a,b).

### 3.2. Polypharmacy and Bone Mineral Density

In unadjusted analyses, lower BMD values were observed among patients with polypharmacy compared with those using fewer medications. In multivariable linear regression models adjusted for age, sex, body mass index, and relevant clinical covariates, polypharmacy remained significantly associated with lower lumbar spine BMD.

In multivariable linear regression analyses with hip BMD as the dependent variable, several medication classes were associated with lower BMD values. Loop diuretics (β = −1.92, 95% CI −2.86 to −0.98, *p* = 0.003), thiazide diuretics (β = −1.28, 95% CI −2.30 to −0.26, *p* = 0.024), NSAIDs (β = −1.65, 95% CI −2.85 to −0.45, *p* = 0.006), and systemic steroid use (β = −1.35, 95% CI −2.45 to −0.25, *p* = 0.018) were associated with lower hip BMD. In contrast, potassium-sparing diuretics (*p* = 0.954), PPIs (*p* = 0.948), antidepressants (*p* = 0.279), and thyroid medications (*p* = 0.202) did not reach statistical significance in their association with hip BMD. Detailed regression results are summarized in Table 2.

Polypharmacy, defined as the concurrent use of five or more medications, was associated with lower lumbar spine BMD (β = −1.75, 95% CI −2.89 to −0.61, *p* = 0.022), while no significant association was observed for hip BMD (β = −0.89, 95% CI −2.09 to 0.31, *p* = 0.633).

Age was strongly associated with lower BMD values in multivariable analyses (β = −2.50, 95% CI −3.20 to −1.80, *p* < 0.001). Sex showed a borderline association with BMD (*p* = 0.052), with lower BMD values observed among female participants.

In medication-specific analyses, thyroid medication use was not associated with hip BMD but was associated with lower lumbar spine BMD (β = −1.45, 95% CI −2.41 to −0.49, *p* = 0.032). The results suggested that thyroid medication use was not a significant correlator of hip BMD but remained significantly associated with lumbar BMD loss.

### 3.3. Non-Linear Associations Between Age and Bone Mineral Density

Given that the association between age and bone mineral density may not be strictly linear, an exploratory quadratic regression model including an age-squared (age^2^) term was evaluated. The quadratic term was not statistically significant for either skeletal site. Model fit statistics indicated higher explained variance for lumbar spine BMD than for hip BMD; however, these findings should be interpreted cautiously and considered exploratory.

## 4. Discussion

In this large cross-sectional analysis of 1155 geriatric patients undergoing routine dual-energy X-ray absorptiometry, polypharmacy and several commonly prescribed medication classes were associated with lower bone mineral density. Associations were more pronounced at the lumbar spine than at the hip, suggesting site-specific differences in observed BMD values across skeletal regions. Beyond individual drug classes, a higher overall medication burden was associated with lower lumbar spine BMD, whereas associations with hip BMD were weaker. These findings support the concept that polypharmacy may reflect broader clinical complexity and multimorbidity that coincide with reduced bone density in older adults, rather than the effects of single medications alone. Previous studies have reported associations between selected drug classes, including thyroid hormone therapy, proton pump inhibitors, and systemic glucocorticoids, and reduced BMD or fracture risk [7,13].

Our findings are consistent with this literature, underlying that polypharmacy was associated with lower bone mineral density, an association that likely reflects underlying frailty and clinical complexity rather than a direct causal effect. In adjusted multivariable analyses, polypharmacy was associated with lower bone mineral density, an association that likely reflects underlying frailty and clinical complexity rather than a direct causal effect. [14,15]. Although causal inferences cannot be drawn from the present cross-sectional design, the observed associations highlight the importance of medication burden as a clinically relevant factor when evaluating bone health in older patients. Given the high prevalence of multimorbidity and complex pharmacotherapy in geriatric populations, systematic medication review and consideration of bone health assessment may be warranted, particularly among patients with extensive medication use.

Our analysis demonstrated a significant association between polypharmacy and lower lumbar BMD (*p* < 0.05), with polypharmacy with stronger associations observed for lumbar spine BMD than for hip BMD (33.4% of the variance and only 8.9% respectively explained). In adjusted multivariable analyses, polypharmacy was associated with lower lumbar spine BMD, whereas unadjusted mean BMD values are presented descriptively in Table 1. Previous studies have consistently linked polypharmacy with an elevated risk of osteoporotic fractures, particularly hip fractures, in community-dwelling older adults and post-fracture populations. Large cohort and claims-based studies have shown that fracture risk increases progressively with the number of prescribed medications, even after adjusting for comorbidity and functional status. Taken together with our findings, the disproportionately stronger association of polypharmacy with lumbar BMD supports the hypothesis that cumulative medication exposure may be preferentially linked to lower trabecular bone density—an area with higher turnover and greater sensitivity to pharmacologic disruptions. This mechanism may partly explain the high vertebral fracture burden observed in multimorbid geriatric patients despite comparable hip BMD [16,17,18].

In addition, the observed patterns in hip BMD and fracture-related outcomes may partly reflect prior hip arthroplasty procedures, which reduce the number of native hips at risk for osteoporotic fracture in very old populations. Previous studies have demonstrated that widespread use of hip arthroplasty may influence observed fracture epidemiology and should be considered when interpreting hip-related bone outcomes in geriatric cohorts [19].

In geriatric populations, polypharmacy is closely linked to multimorbidity, and medication use often reflects underlying disease patterns and severity rather than direct pharmacological effects. Although our models adjusted for selected individual comorbidities, this approach does not fully capture the complexity or severity of multimorbidity.

It is therefore plausible that some observed associations, particularly those involving cardiovascular medications such as thiazide diuretics, reflect confounding by indication. In this context, medications may serve as proxy markers of disease burden, frailty, or advanced clinical complexity rather than causal drivers of reduced bone mineral density. While multimorbidity cluster analyses have been proposed as a means to better characterize disease patterns in older adults, such approaches were beyond the scope of the present retrospective analysis.

The observed discordance between lumbar spine and hip BMD findings warrants careful interpretation. In very old populations, lumbar spine measurements are susceptible to degenerative changes, such as osteophytes, vertebral deformities, and vascular calcifications, which typically lead to spuriously elevated rather than reduced BMD values. The finding that polypharmacy was associated with lower lumbar spine BMD despite this potential upward bias suggests that the observed association is unlikely to be solely driven by degenerative artifact. From a biological perspective, trabecular-rich bone at the lumbar spine exhibits higher turnover and greater sensitivity to pharmacologic and metabolic influences than cortical-dominant bone at the hip. This may partly explain why associations with medication burden were more pronounced at the lumbar spine. In addition, the lower explained variance observed in hip BMD models compared with lumbar spine models may reflect greater measurement variability or residual confounding at the hip site in this very old inpatient cohort. The retrospective nature of the study precluded calculation of site-specific precision metrics (CV%, LSC) and systematic exclusion of degenerative artifacts, which may have introduced some imprecision in BMD estimates and contributed to site-specific differences in model performance, particularly at the lumbar spine.

The observed differences of L-Thyroxin on lumbar BMD were statistically significant, while its impact on hip BMD remained non-significant. This finding suggests that thyroid hormone supplementation may preferentially affect trabecular-rich bone sites [20,21]. Excess thyroid hormone levels have been associated with increased bone turnover and osteoclastic resorption, with reduced BMD and heightened fracture risk, particularly in the vertebrae [22,23]. Existing evidence suggests that clinically relevant skeletal effects of levothyroxine are primarily observed in the context of suppressive or excessive dosing, whereas adequately titrated replacement therapy appears to have minimal impact on bone mineral density [24]. Levothyroxine showed in our study a site-specific association with lumbar but not hip BMD, consistent with prior reports indicating that excess thyroid hormone preferentially affects trabecular bone and increases vertebral fracture risk even in biochemically euthyroid patients with low-normal TSH.

Similar site-specific patterns have been reported in previous work examining subclinical hyperthyroidism and levothyroxine overtreatment [25]. The association between levothyroxine use and lower lumbar spine BMD should be interpreted cautiously, as data on thyroid function tests and treatment intensity were not available for all patients. It is therefore not possible to distinguish adequately titrated replacement therapy from potential over-replacement, which may partly explain the observed site-specific association.

Among the various drug classes analysed, loop diuretics showed a strong negative impact on hip BMD, followed by thiazide diuretics, NSAIDs, and steroids. The negative impact of loop diuretics is particularly concerning, as these medications promote calcium excretion, potentially leading to bone demineralization over time [26,27]. While thiazide diuretics have been previously associated with a protective effect on BMD due to their calcium-sparing properties, still demonstrated a negative association in this advanced-age study, possibly reflecting confounding by indication or frailty. Thiazide diuretics have been reported to exert calcium-sparing effects and are generally considered neutral or protective with respect to bone health. The inverse association observed in this cohort likely reflects confounding by indication, as thiazide use may serve as a marker of cardiovascular disease burden, frailty, or multimorbidity rather than a direct adverse skeletal effect.

Steroid use was significantly associated with reduced hip BMD, supporting the well-established link between chronic glucocorticoid use and osteoporosis. The association of steroid use with lower BMD aligns with extensive evidence linking chronic glucocorticoid exposure to reduced bone formation and increased resorption. However, in our population, steroid use may also indicate underlying inflammatory or autoimmune disease, which itself can contribute to BMD loss. The findings further emphasize that patients using both diuretics and PPIs had significantly lower hip BMD compared to those taking either drug alone [28,29]. This raises concerns regarding the concurrent use of these medications, as PPIs are known to interfere with calcium absorption by reducing gastric acidity, further compounding the effects of diuretics.

The interaction between PPIs, diuretics and bone health is particularly relevant. Meta-analyses and multiple observational studies have documented a modest but consistent increase in hip fracture risk among long-term PPI users, although effects on BMD alone are inconsistent [30,31]. PPIs impair calcium absorption through hypochlorhydria, and this effect may become clinically significant when combined with loop diuretics, which increase renal calcium loss [32]. Our finding that PPI + diuretic co-use was significantly associated with lower hip BMD supports the concept of additive or synergistic skeletal effects arising from concurrent disturbances in calcium homeostasis. Thus, potassium-sparing diuretics and PPIs alone may be not significantly associated with changes in BMD when evaluated independently; however, their combination with other medications, particularly diuretics, suggests a cumulative impact on bone health [33,34].

These findings emphasize the importance of carefully weighing medication choices in older adults, as several drug classes commonly used in this age group showed associations with lower BMD. Because many medications may function as markers of underlying diseases rather than direct causes of bone loss, deprescribing alone is unlikely to address osteoporosis risk and may, in some cases, be inappropriate. Instead, clinicians should critically evaluate the necessity, dosage, and selection of medication groups—particularly in patients with multimorbidity—while simultaneously considering the underlying conditions that contribute to both medication use and reduced BMD [35]. Regular BMD monitoring is particularly recommended for individuals taking multiple osteoporosis-risk medications, including L-Thyroxin, PPIs, steroids, and diuretics [36]. Furthermore, calcium and vitamin D supplementation should be considered in high-risk individuals to help counteract medication-induced bone loss [37,38].

Frailty represents a central construct linking polypharmacy, multimorbidity, and adverse skeletal outcomes in older adults. Frail individuals are more likely to receive multiple medications, experience reduced mobility and nutritional deficits, and exhibit chronic inflammatory states that may adversely affect bone metabolism [39,40]. Although frailty was not directly measured in the present study, the advanced age and inpatient status of the cohort suggest a high underlying frailty burden. The absence of a standardized frailty assessment limits the ability to disentangle whether polypharmacy acts as an independent risk factor for reduced bone mineral density or primarily reflects underlying frailty and disease severity [41]. It is therefore plausible that polypharmacy functions as a proxy marker of frailty-related clinical complexity rather than a direct causal driver of bone loss. Future prospective studies in geriatric populations should incorporate validated frailty instruments to clarify the interplay between medication burden, frailty trajectories, and skeletal health [42].

The present findings should be interpreted in light of potential selection mechanisms. The study population consisted of geriatric inpatients undergoing DXA as part of routine clinical evaluation, most often due to suspected osteoporosis, prior fractures, or complex multimorbidity. Consequently, the cohort likely represents individuals at higher baseline risk for low bone mineral density compared with community-dwelling older adults. In addition, survivor bias may be present, as all participants had survived to a very advanced age. This may attenuate or modify observed associations between medication burden and bone mineral density. While these factors limit generalizability to healthier older populations, they also reflect real-world clinical practice in geriatric inpatient settings, where polypharmacy and osteoporosis frequently coexist.

A major strength of this study is the large sample size, which allowed for detailed analyses of medication use patterns and their associations with bone mineral density across different skeletal sites. Bone mineral density was assessed using dual-energy X-ray absorptiometry (DXA), providing standardized and reliable measurements at both the lumbar spine and hip. In addition, the inclusion of multiple medication classes alongside an assessment of overall medication burden enabled a broad evaluation of polypharmacy in a geriatric inpatient population.

Several limitations should also be acknowledged. First, the cross-sectional study design precludes causal inference and limits interpretation to observed associations between medication use, polypharmacy, and bone mineral density. Second, although analyses were adjusted for key confounders such as age, sex, and body mass index, residual confounding cannot be excluded. Information on additional factors influencing bone health, including dietary calcium intake, physical activity, and genetic susceptibility, was not available. Another important limitation of this study is the inability to reliably identify and exclude patients with prior or ongoing osteoporosis-specific pharmacological treatment. In very old and multimorbid inpatient populations, treatment histories often extend over many years and are incompletely documented in routine electronic medical records. Consequently, some participants may have had pharmacologically modified BMD values, which could attenuate or obscure associations between medication burden and bone mineral density. However, excluding all patients with any history of osteoporosis therapy would have substantially reduced the sample size and compromised the representativeness of real-world geriatric care. This limitation should be considered when interpreting the magnitude of observed associations, which likely reflect real-world clinical complexity rather than untreated bone health trajectories.

Finally, an important limitation of this study is the lack of detailed information on medication dose, duration, and adherence. For several drug classes, including levothyroxine and systemic glucocorticoids, skeletal effects are known to be dose- and duration-dependent. The absence of these data may have led to misclassification of exposure, potentially conflating short-term or low-dose users with long-term or high-dose users. Consequently, the observed associations should be interpreted as reflecting real-world medication exposure patterns rather than causal effects of specific treatment regimens. In addition, the lack of DXA precision metrics (CV% and least significant change) and the inability to systematically exclude degenerative artifacts may have introduced some imprecision in BMD estimates, particularly at the lumbar spine, and should be considered when interpreting site-specific associations.

## 5. Conclusions

This study provides evidence that polypharmacy is significantly associated with lower lumbar BMD, with specific drug classes—including L-Thyroxin, PPIs, steroids, and diuretics—demonstrating notable negative associations with bone health. The combination of diuretics and PPIs, in particular, was associated with significant reductions in hip BMD. Given the widespread use of these medications in older adults, these findings underscore the need for enhanced osteoporosis screening and preventive strategies in high-risk populations. Future prospective studies should further explore the long-term impact of polypharmacy on bone health by integrating standardized frailty assessments, such as the Clinical Frailty Scale, FRAIL scale, or Edmonton Frail Scale, alongside detailed evaluation of medication dosage, treatment duration, and potential drug–drug interactions, in order to disentangle medication-related effects from underlying clinical frailty.

## Figures and Tables

**Table 1:** Baseline characteristics of the study population stratified by polypharmacy status. Table 1 presents descriptive baseline characteristics stratified by polypharmacy status; inferential comparisons are provided in unadjusted and multivariable analyses.

Characteristic	Total Cohort (n = 1155)	<5 Medications (n = 410)	≥5 Medications (n = 745)
Age, years (mean ± SD)	85.0 ± 7.1	84.7 ± 7.9	85.2 ± 6.6
Female sex, n (%)	925 (80.1%)	334 (81.5%)	591 (79.3%)
Fracture at index hospitalization, n (%) *	607 (52.6%)	218 (47.7%)	389 (52.2%)
Barthel Index (mean ± SD)	—	34.1 ± 18.1	31.3 ± 16.7
Lumbar spine BMD, T-score (mean ± SD)	—	−1.47 ± 1.91	−1.25 ± 1.76
Total hip BMD, T-score (mean ± SD)	—	−1.96 ± 1.10	−1.97 ± 1.23
Vitamin D available, n (%)	—	325 (79.2%)	655 (89.9%)

* Fracture status refers to clinically documented fragility fractures at the time of index hospitalization and does not represent lifetime fracture history. Fracture data were available for a subset of patients.

**Table 2:** Multivariable linear regression models for lumbar spine and total hip BMD. (**A**). Lumbar spine BMD. (**B**). Total hip BMD.

A. Lumbar Spine BMD			
Predictor	β Coefficient	95% CI	*p*-Value
Age (per year)	−2.50	−3.20 to −1.80	<0.001
Polypharmacy (≥5 meds)	−1.75	−2.89 to −0.61	0.022
Levothyroxine use	−1.45	−2.41 to −0.49	0.032
Glucocorticoid use	−1.35	−2.45 to −0.25	0.057
Model R^2^ = 0.334			
**B. Total Hip BMD**			
**Predictor**	**β Coefficient**	**95% CI**	***p*** **-Value**
Age (per year)	−2.10	−2.80 to −1.40	<0.001
Polypharmacy (≥5 meds)	−0.89	−2.09 to 0.31	0.633
Loop diuretics	−1.92	−2.86 to −0.98	0.003
Thiazide diuretics	−1.28	−2.30 to −0.26	0.024
NSAIDs	−1.65	−2.85 to −0.45	0.006
Systemic steroids	−1.35	−2.45 to −0.25	0.018
Model R^2^ = 0.089			

## Data Availability

The data presented in this study are not publicly available due to ethical and legal restrictions, as they contain sensitive clinical information derived from routinely collected electronic medical records. Anonymized data may be made available from the corresponding author upon reasonable request and subject to approval by the local ethics committee.

## Supplementary Materials

The following supporting information can be downloaded at: https://www.mdpi.com/article/10.3390/jcm15031197/s1, Figure S1: STROBE flow diagram of patient identification, exclusions, and final analytical sample; Table S1: STROBE Checklist for Cross-Sectional Studies.
