# Supplementary material for: Association of Polypharmacy and Bone Mineral Density: A Cross-Sectional Analysis of Geriatric Inpatients in Germany
Source: J Clin Med. 2026 Feb 3;15(3):1197. doi: 10.3390/jcm15031197 (PMC12898433; doi:10.3390/jcm15031197)
Supplement: Supplementary file 1 [file jcm-15-01197-s001.zip › jcm-4105699-supplementary.pdf]

Supplementary Material

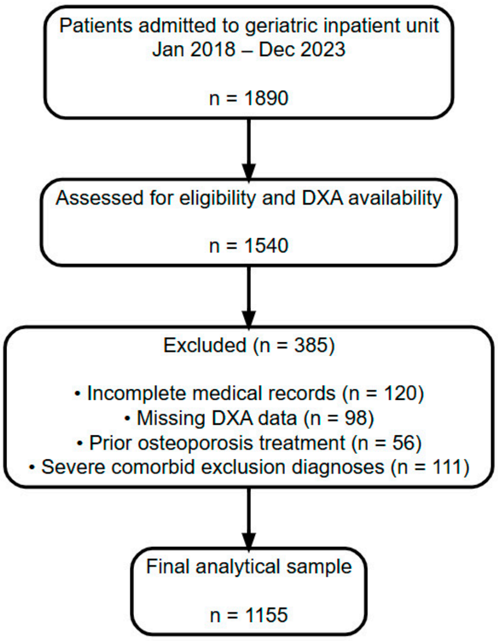

Supplementary Figure S1. STROBE flow diagram of patient identification, exclusions, and final analytical sample.

**STROBE Item**	**Recommendation**	**Location in Manuscript**
**Title & Abstract**		
1a	Indicate the study design in the title or abstract	Title; Abstract (Methods)
1b	Provide an informative and balanced summary	Abstract
**Introduction**		
2	Scientific background and rationale	Introduction, paragraphs 1–3
3	Specific objectives and hypotheses	Introduction, final paragraph
**Methods**		
4	Key elements of study design	Materials and Methods—Study Design and Population
5	Setting, locations, and relevant dates	Materials and Methods—Data Collection and Patient Selection
6a	Eligibility criteria and participant selection	Materials and Methods—Inclusion and Exclusion Criteria
6b	Matching criteria (if applicable)	Not applicable
7	Definition of outcomes, exposures, confounders	Materials and Methods—Bone Mineral Density Assessment; Statistical Analysis
8	Data sources and measurement methods	Materials and Methods—Data Collection and Patient Selection; Bone Mineral Density Assessment

STROBE Item	Recommendation	Location in Manuscript
9	Methods to address potential bias	Materials and Methods—Inclusion and Exclusion Criteria; Statistical Analysis
10	Study size rationale	Materials and Methods—Study Design and Population
11	Handling of quantitative variables	Materials and Methods—Statistical Analysis
12a	Statistical methods, including confounding control	Materials and Methods—Statistical Analysis
12b	Subgroup or interaction analyses	Materials and Methods—Statistical Analysis
12c	Handling of missing data	Materials and Methods—Statistical Analysis
12d	Sampling strategy	Not applicable
12e	Sensitivity analyses	Materials and Methods—Statistical Analysis
**Results**		
13a	Numbers of participants at each stage	Results—Study Population; Figure (STROBE flow diagram)
13b	Reasons for non-participation	Results—Study Population
13c	Flow diagram	Figure X (STROBE flow diagram)
14a	Participant characteristics	Results—Study Population; Table 1
14b	Missing data for variables	Results; Table 1
15	Outcome data	Results—Associations between medication use and BMD
16a	Unadjusted and adjusted estimates with precision	Results—Polypharmacy and bone mineral density; Table 2
16b	Category boundaries for variables	Materials and Methods—Statistical Analysis
16c	Translation to absolute risk	Not applicable
**Discussion**		
17	Key results in relation to objectives	Discussion, paragraphs 1–2
18	Study limitations	Discussion—Strengths and Limitations
19	Interpretation considering limitations	Discussion
20	Generalisability	Discussion
**Other Information**		
21	Funding source and role of funders	Funding statement

Supplementary Table S1. STROBE Checklist for Cross-Sectional Studies
